# ESCRT-III is recruited by human herpesvirus 6A nuclear egress complex to promote nuclear egress of the nucleocapsid

**DOI:** 10.1128/jvi.00844-25

**Published:** 2025-08-19

**Authors:** Aila Gulijiahani, Jun Arii, Vuk Isakovic, Jing Rin Huang, Yasushi Kawaguchi, Yasuko Mori

**Affiliations:** 1 Division of Clinical Virology, Center for Infectious Diseases, Kobe University Graduate School of Medicine, Kobe, Hyogo, Japan; 2 Division of Molecular Virology, Department of Microbiology and Immunology, The Institute of Medical Science, The University of Tokyo 592607 , Minato-ku, Tokyo, Japan; 3 Department of Infectious Disease Control, International Research Center for Infectious Diseases, The Institute of Medical Science, The University of Tokyo 596903 , Minato-ku, Tokyo, Japan; 4 Research Center for Asian Infectious Diseases, The Institute of Medical Science, The University of Tokyo 592662 , Minato-ku, Tokyo, Japan; 5 Pandemic Preparedness, Infection and Advanced Research Center, The University of Tokyo 13143, Minato-ku, Tokyo, Japan; University of Virginia, Charlottesville, Virginia, USA

**Keywords:** ESCRT-III, nuclear egress, HHV-6, herpesviruses

## Abstract

Herpesviruses replicate their genomes and package them into capsids within the host cell nucleus. These capsids must then translocate from the nucleus to the cytoplasm through a process designated nuclear egress. The virus-encoded nuclear egress complex (NEC), consisting of a nuclear matrix protein and a nuclear membrane protein, plays a crucial role in this process. Although the role of NEC for nucleo-cytoplasmic transport of capsids is conserved in *Herpesviridae*, some of the binding partners of the NEC components are specific for individual viruses. The NECs of alpha- and gammaherpesviruses recruit the Endosomal Sorting Complex Required for Transport III (ESCRT-III) to the inner nuclear membrane for efficient nuclear egress of capsids. In contrast, the role of ESCRT-III for nuclear egress of betaherpesviruses, including human cytomegalovirus (HCMV) and human herpesvirus 6A (HHV-6A), has not been elucidated. Here, we show that ESCRT-III is recruited to the nuclear rim in cells expressing the NEC of HCMV or of HHV-6A. Inhibition of ESCRT-III impaired HHV-6A replication and nuclear egress of the capsids. Mechanistically, ESCRT-III adaptor ALIX interacts with HHV-6A NEC membrane protein U34 and thus contributes to HHV-6A replication. From these observations, we conclude that, like at least some alpha- and gammaherpesviruses, HHV-6A NEC recruits ESCRT-III through ALIX to promote viral capsid nuclear egress.

**IMPORTANCE:**

ESCRT-III performs reverse-topology scission involved in many diverse cellular processes, including cytokinesis, endosome maturation, autophagy, membrane repair, and viral budding. Nucleo-cytoplasmic transport of herpesvirus capsids requires scission at the inner nuclear membrane. In alpha- and gammaherpesviruses, this process requires ESCRT-III, but it is not known whether this is also the case for betaherpesviruses. Here, we show that ESCRT-III is also important for nuclear egress of capsids of the betaherpesvirus human herpesvirus 6A. These results imply that ESCRT-III-mediated inner nuclear membrane scission is a conserved feature in the virion maturation process of *Herpesviridae*. Our findings thus suggest that ESCRT-III is a potential therapeutic target also for betaherpesvirus infections.

## INTRODUCTION

There are nine species of human herpesviruses that cause lifelong persistent infection. The *Herpesviridae* family is subdivided into *Alphaherpesvirinae*,* Betaherpesvirinae*, and *Gammaherpesvirinae* subfamilies, based on their molecular and biological properties. Herpesviruses replicate their genomes and package them into capsids within the host cell nucleus (1–3). These nucleocapsids must then be transported to the cytoplasm through a process designated nuclear egress. First, the nascent nucleocapsids bud at the inner nuclear membrane (INM) to form primary virions in the perinuclear space. Next, the envelopes of primary virions fuse with the outer nuclear membrane (ONM), releasing the nucleocapsids into the cytoplasm (1–3). In the cytoplasm, capsids bud into vesicles derived from trans-Golgi networks or endosomes in a process called secondary envelopment. Finally, enveloped virions are released from the cells through an exocytotic pathway (1).

**Fig 1:**
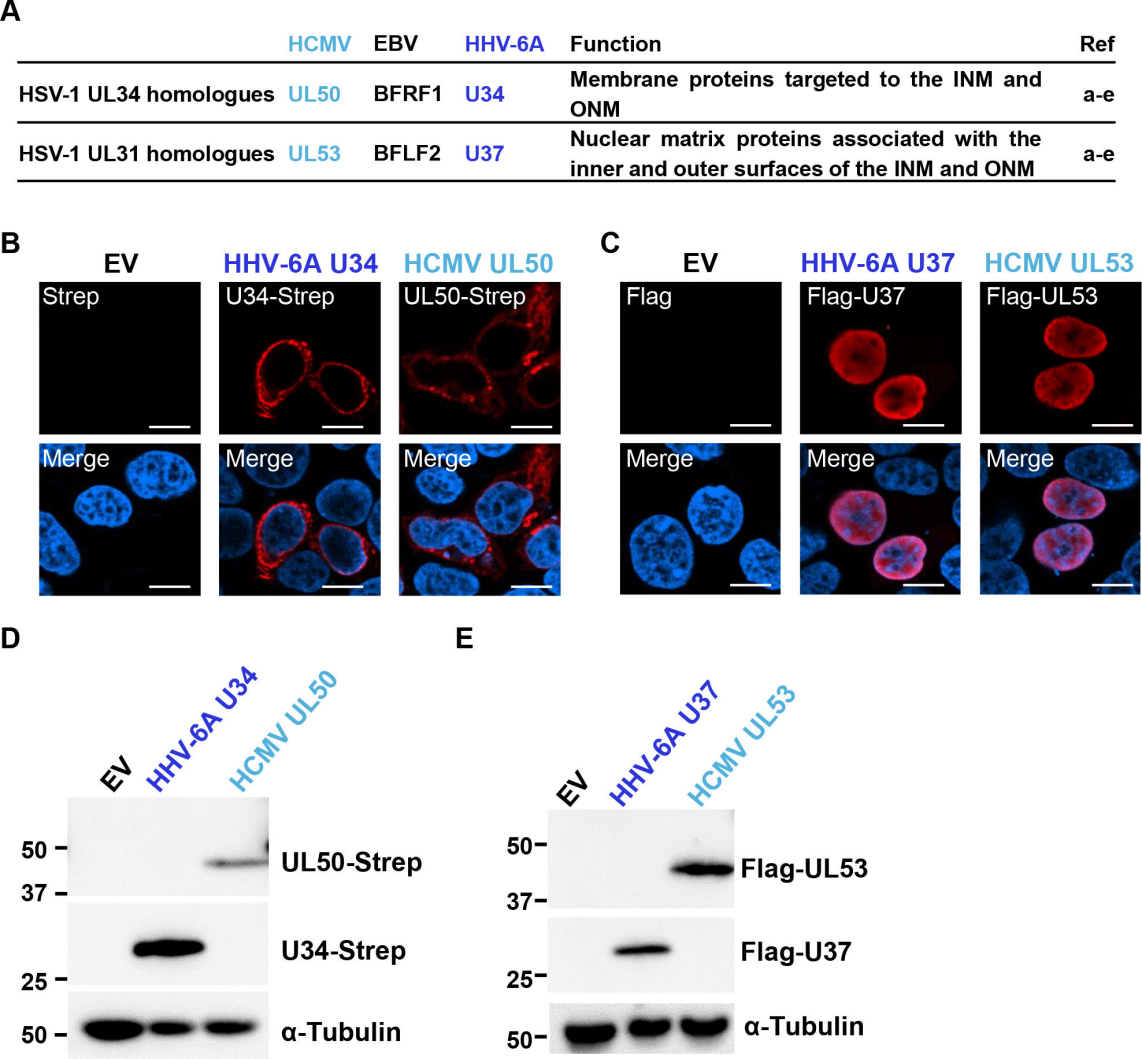
Exogenous expression of the NEC components from human herpesvirus 6A (HHV-6A) and HCMV. (**A**) Description of the NEC components in each virus. (a) (4); (b) (7); (c) (6); (d) (5); and (e) (8). (B to E) HEK293T cells were transfected with plasmids encoding HHV-6A U34-Strep, HCMV UL50-Strep, Flag-HHV-6A U37, or Flag-HCMV UL53. After 48 hours, the cells were harvested, fixed, and analyzed by confocal microscopy (**B and C**) or immunoblotting (**D and E**). EV, empty vector. Bar, 10 µm.

The nuclear egress complex (NEC), composed of two viral proteins, plays a crucial role in the nucleocytoplasmic transport of newly-assembled nucleocapsids (1–3). In the alphaherpesvirus herpes simplex virus 1 (HSV-1), these proteins are designated UL31 and UL34, while in the betaherpesvirus human cytomegalovirus (HCMV) and gammaherpesvirus Epstein-Barr virus (EBV), they are designated UL50 and UL53, and BFRF1 and BFLF2, respectively (Fig. 1A). The UL34 homologs are membrane proteins targeted to both the INM and the ONM (4–8), whereas the UL31 homologs are nuclear matrix proteins held in close apposition to the inner and outer surfaces of the INM and ONM through interaction with individual nuclear membrane proteins (4–8). Conversely, INM localization of the nuclear membrane protein is enhanced in the presence of the nuclear matrix protein (4–8). Molecular biological and biochemical analyses revealed that the HSV-1 NEC recruits capsids into vesicles and mediates scission of the INM to finalize envelopment (4, 9–12). The solved crystal structures of the core region of the NEC from herpesviruses reveal the highly conserved features of this complex (13–19). However, it is not known whether the NEC is completely conserved, due to the diversity of amino acid sequences of NEC components.

The Endosomal Sorting Complex Required for Transport (ESCRT)-III is part of the cell membrane remodeling machinery (20, 21). ESCRT-III employs polymer formation to catalyze inside-out membrane fission processes involved in many diverse cellular processes, including budding of endosomal vesicles and enveloped viruses as well as cytokinesis (20, 21). ESCRT-III activity is considered to be crucial for secondary envelopment of herpesviruses (22–24). In addition, NECs from alpha- and gammaherpesviruses recruit ESCRT-III to the nuclear membrane to mediate scission of the INM for capsid nucleocytoplasmic transport (25–28), utilizing the ALIX adaptor protein to link ESCRT-III and the NEC (25, 26, 28). In contrast, a role for ESCRT-III in nuclear egress of betaherpesviruses has not been shown, although it is known that ESCRT-III is not critical for HCMV replication (29, 30).

Human herpesvirus 6A (HHV-6A) is a lymphotropic virus belonging to the *Roseolovirus* genus within the *Betaherpesvirinae* subfamily (31). HHV-6A is frequently found in patients with neuro-inflammatory diseases such as multiple sclerosis and Hashimoto’s thyroiditis (32, 33). The roles of HHV-6A NEC components have not been well-studied. Recently, we found that the HHV-6A nuclear matrix protein U37 specifically induces a heat shock response through binding to heat shock factor 1 to promote viral replication (8). In the present study, we further analyze the role of HHV-6A NEC and show that ESCRT-III machinery is also important for nuclear egress of HHV-6A capsids and, therefore, for replication of this virus.

## RESULTS

### NEC components from HHV-6A and HCMV interact with each other

**Fig 2:**
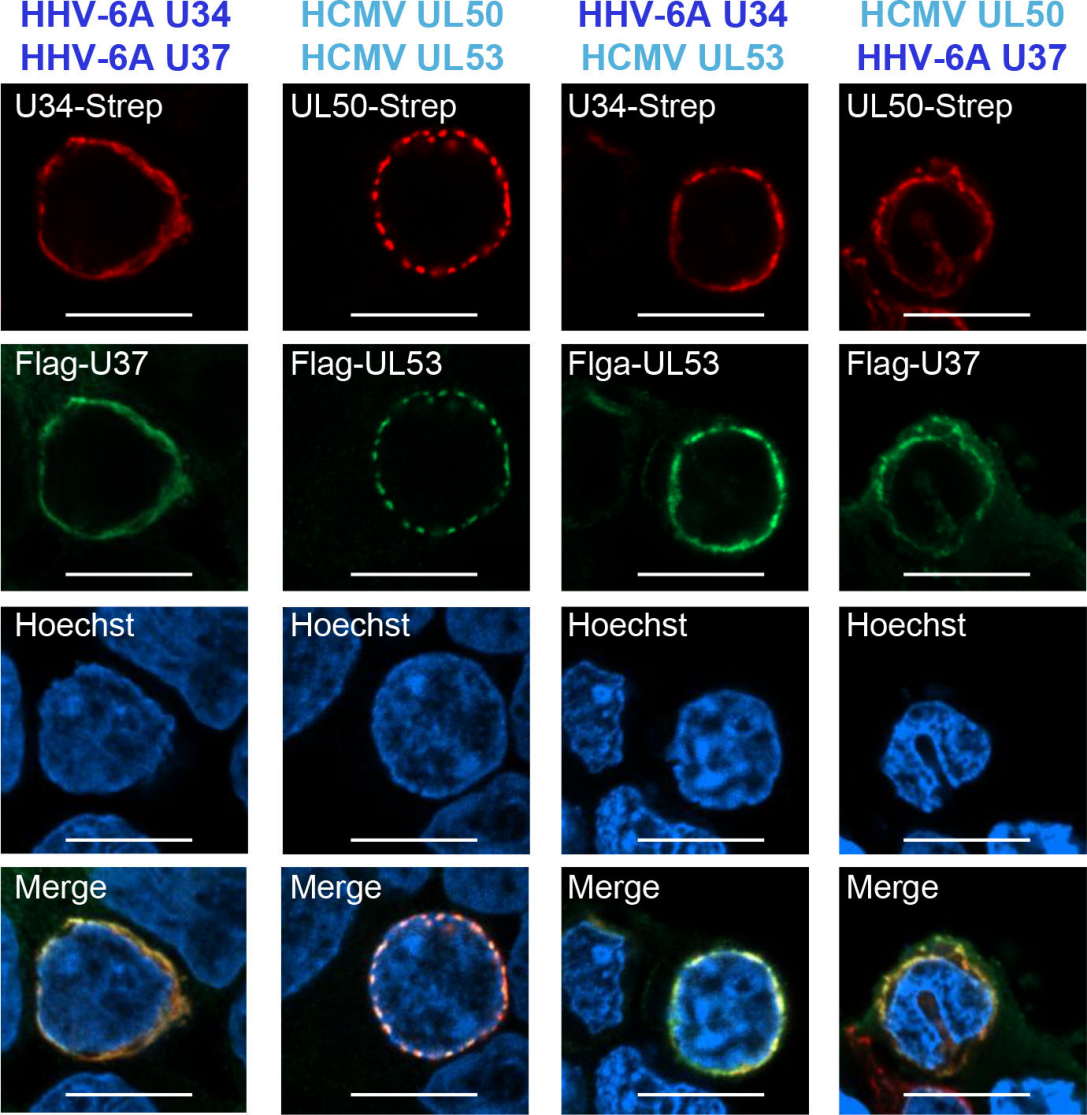
Co-localization of NEC components from HHV-6A and HCMV. HEK293T cells were co-transfected with the indicated combinations of plasmids encoding NEC components from HHV-6A or HCMV. After 48 hours, the cells were fixed and stained with antibodies specific for the Strep and Flag tags and visualized by confocal microscopy. Bar, 10 µm.

To investigate the roles of HHV-6A NEC, we first analyzed the subcellular localization of these proteins by transient expression of HHV-6A U34-Strep, HCMV UL50-Strep, Flag-HHV-6A U37, or Flag-HCMV UL53 in HEK293T cells. Forty-eight hours after transfection, the cells were analyzed by immunoblotting and fluorescence assays. As expected, HHV-6A U34-Strep and HCMV UL50-Strep were detected in cytoplasmic membranous structures or the nuclear rim, whereas Flag-HHV-6A U37 and Flag-HCMV UL53 were present in the nucleoplasm (Fig. 1B and C) as previously reported (8, 34). Immunoblotting confirmed the expression of each NEC component in transfected cells (Fig. 1D and E). To determine whether HCMV and HHV-6A NEC components interact, these plasmids were co-transfected into HEK293T cells. After 48 hours, the cells were analyzed by fluorescence assays. As previously reported (8, 34), Flag-HHV-6A U37 and Flag-HCMV UL53 co-localized with HHV-6A U34-Strep or HCMV UL50-Strep at the nuclear rim, respectively (Fig. 2). Similarly, HCMV UL53 or HHV-6A U37 was re-localized to the nuclear rim in the presence of HHV-6A U34 or HCMV UL50, respectively (Fig. 2), indicating that HHV-6A and HCMV NEC components do interact. These observations suggest that the structures and at least some functions of NECs are conserved among betaherpesviruses.

### HHV-6A NEC recruits ESCRT-III protein CHMP4B to the nuclear rim

**Fig 3:**
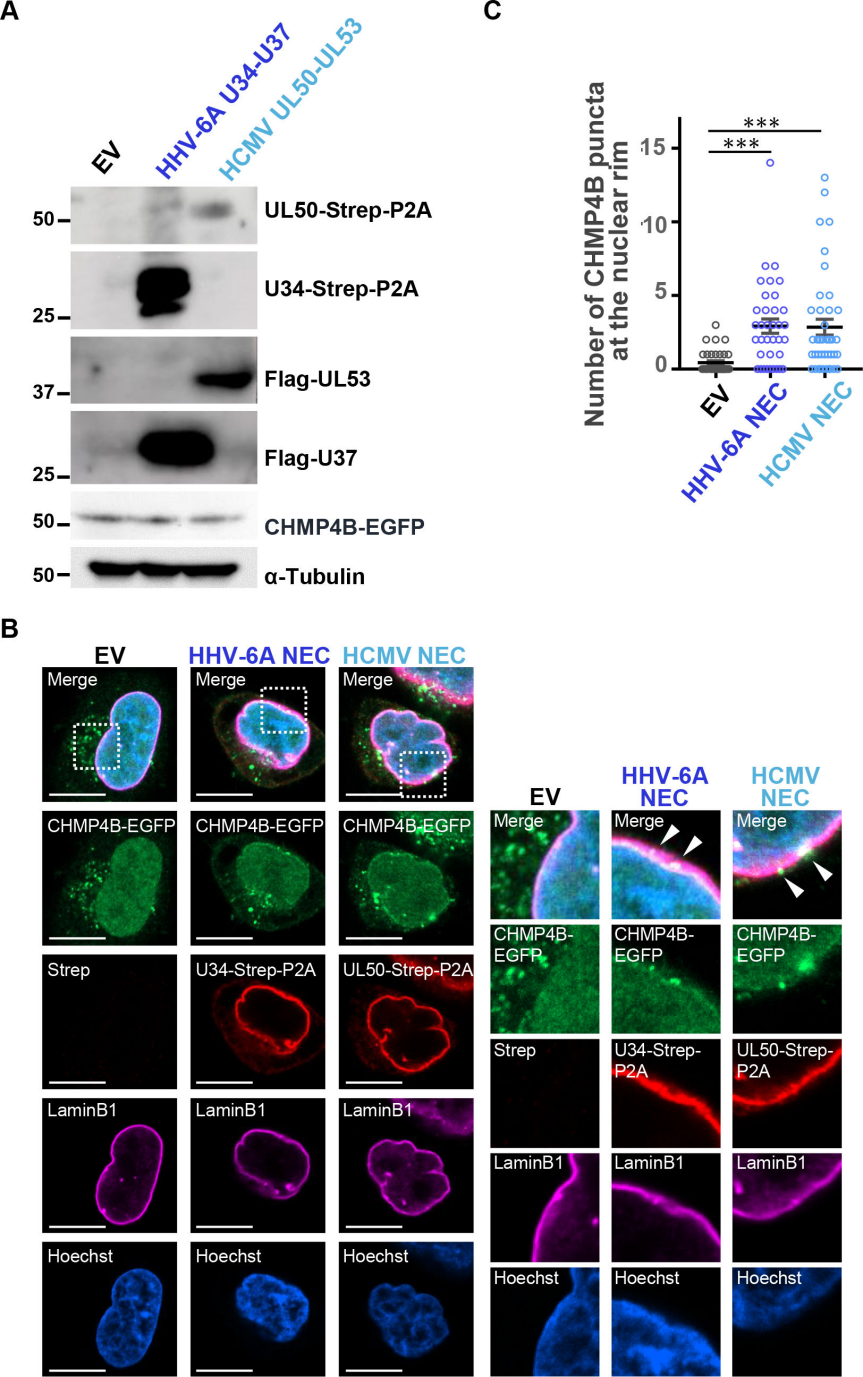
ESCRT-III protein CHMP4B is recruited to the nuclear rim by NEC components of HHV-6A and HCMV. HeLa-CHMP4B-EGFP cells were transfected with plasmids encoding HHV-6A U34-Strep/Flag-U37 or HCMV UL50-Strep/Flag-UL53. After 48 hours, the cells were analyzed by immunoblotting (**A**) and immunofluorescence (**B**) with the indicated antibodies. Each image in the right-hand panels is the magnified image of the boxed area in the left-hand panels. Arrowheads indicate the accumulation of CHMP4B. EV, empty vector. Bar, 10 µm. (**C**) The CHMP4B-EGFP-positive punctate structures at the nuclear rim in the experiment in (**B**) were quantified. Data are shown as the mean ± standard errors for 37 (EV), 36 (HHV-6A NEC), or 40 (HCMV NEC) cells and are representative of three independent experiments (***, *P* < 0.001, Tukey’s test).

**Fig 4:**
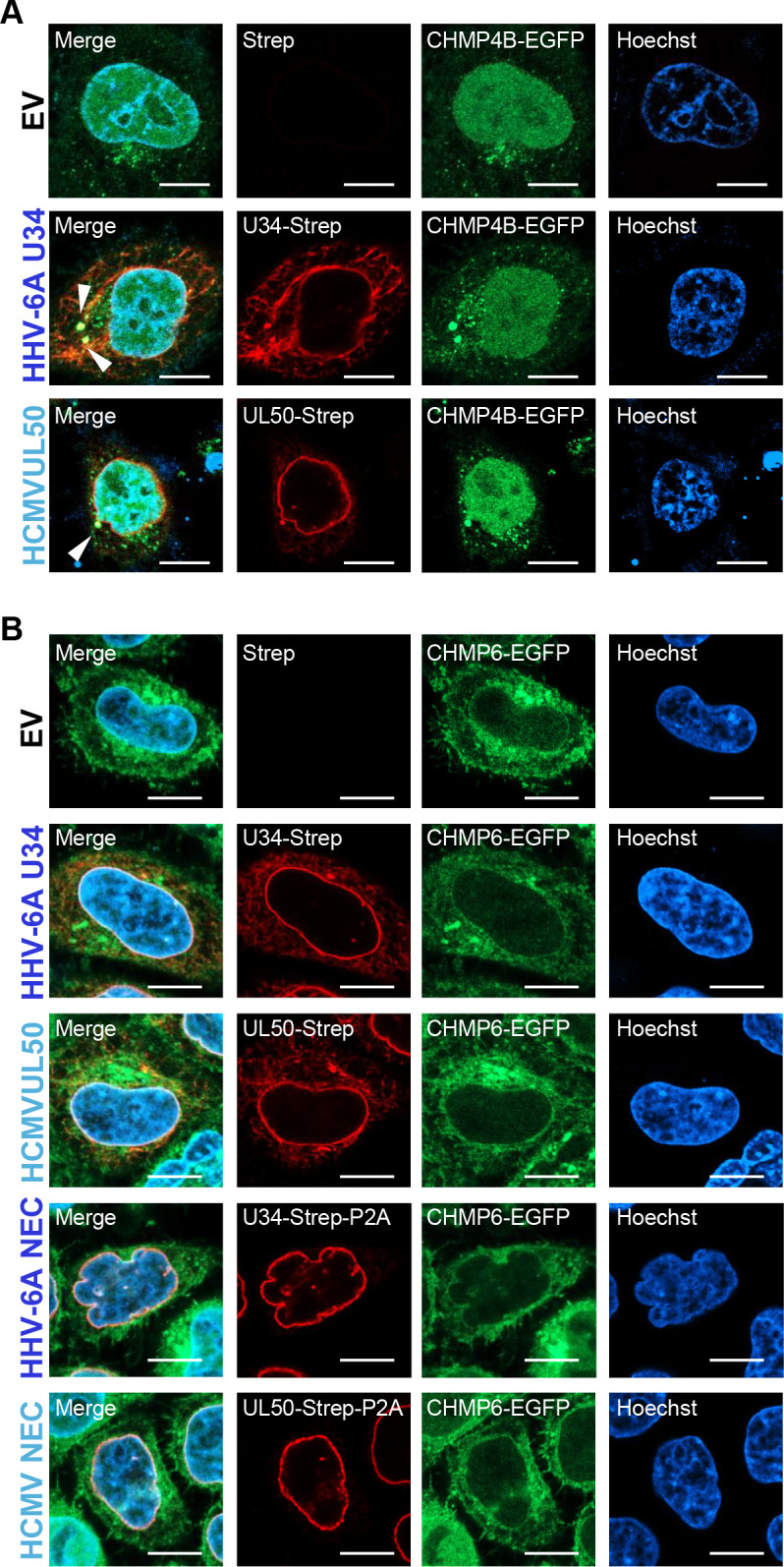
HHV-6A U34 and HCMV UL50 accumulate ESCRT-III protein CHMP4B. (**A**) HeLa-CHMP4B-EGFP cells were transfected with plasmids encoding HHV-6A U34-Strep or HCMV UL50-Strep. After 48 hours, the cells were analyzed by immunofluorescence with the anti-Strep antibody and Hoechst. Arrowheads indicate the accumulation of CHMP4B. (**B**) HeLa-CHMP6-EGFP cells were transfected with plasmids encoding HHV-6A U34-Strep, HHV-6A U34-Strep/Flag-U37, HCMV UL50-Strep, or HCMV UL50-Strep/Flag-UL53. After 48 hours, the cells were analyzed by immunofluorescence with the anti-Strep antibody and Hoechst. EV, empty vector. Bar, 10 µm.

It has been reported that NEC from alpha- and gammaherpesviruses recruit ESCRT-III to the nuclear rim (25, 26), whereas the role of ESCRT-III on nuclear egress of betaherpesviruses has not been established. We therefore investigated whether the HCMV and HHV-6A NECs recruit ESCRT-III. To this end, HeLa-CHMP4B-EGFP cells stably expressing a fusion protein of the ESCRT-III component CHMP4 and EGFP (26, 35) were transfected with plasmids for co-expression of HHV-6A U34-Strep/Flag-HHV-6A U37 or for HCMV UL50-Strep/Flag-HCMV UL53. The former encodes the fusion protein of HHV-6A U34-Strep, P2A self-cleaving peptides, and HHV-6A Flag-U37 to produce U34-Strep-P2A and Flag-U37; the latter similarly produces UL50-Strep-P2A and Flag-UL53 (8). Immunoblotting of the lysate of the transfected cells after 48 hours showed that these cells produced the NEC components separately (Fig. 3A). The cells were then stained with anti-Strep and anti-Lamin B1 antibodies and analyzed by immunofluorescence. As previously reported, CHMP4B-EGFP was observed throughout the nucleus and cytoplasm (Fig. 3B). In cells transfected with each co-expression plasmid, U34-Strep or UL50-Strep localized to the nuclear rim. CHMP4B-EGFP formed punctate structures along the nuclear membrane, marked by Lamin B1, and colocalized with U34-Strep or UL50-Strep. Quantification of CHMP4B-positive puncta at the nuclear envelope revealed a significant increase in NEC-expressing cells compared to controls (Fig. 3C). These results suggest that the NEC recruits ESCRT-III machinery to the nuclear rim and that this ability is conserved among *Herpesviridae*. Of note, the expression of either U34-Strep or UL50-Strep alone was sufficient to induce punctate structures of CHMP4B-EGFP on the domains composed of U34 or UL50 (Fig. 4A), consistent with previous observations on HSV-1 and EBV (25, 28).

CHMP6 is a component of the ESCRT-III complex and functions to link the upstream ESCRT-II complex with CHMP4 proteins. Expression of HHV-6A or HCMV NEC did not alter the subcellular localization of CHMP6 (Fig. 4B), suggesting that the recruitment of CHMP4B by these NECs is specific and that HHV-6A and HCMV NECs activate ESCRT-III independently of ESCRT-II. Consistently, expression of U34 or UL50 alone also did not affect CHMP6 localization, further supporting the specificity of CHMP4B recruitment by the complete NEC. Although CHMP6 was used here as a reference for non-recruitable ESCRT-III components, we acknowledge that it is not an ideal negative control, as it exhibits partial nuclear membrane localization even in cells transfected with an empty vector. Nevertheless, quantitative analysis revealed a significant increase in the number of CHMP4B-positive dots at the nuclear rim in NEC-expressing cells (Fig. 3C), supporting the conclusion that NEC components promote the recruitment of CHMP4B to the nuclear envelope.

**Fig 5:**
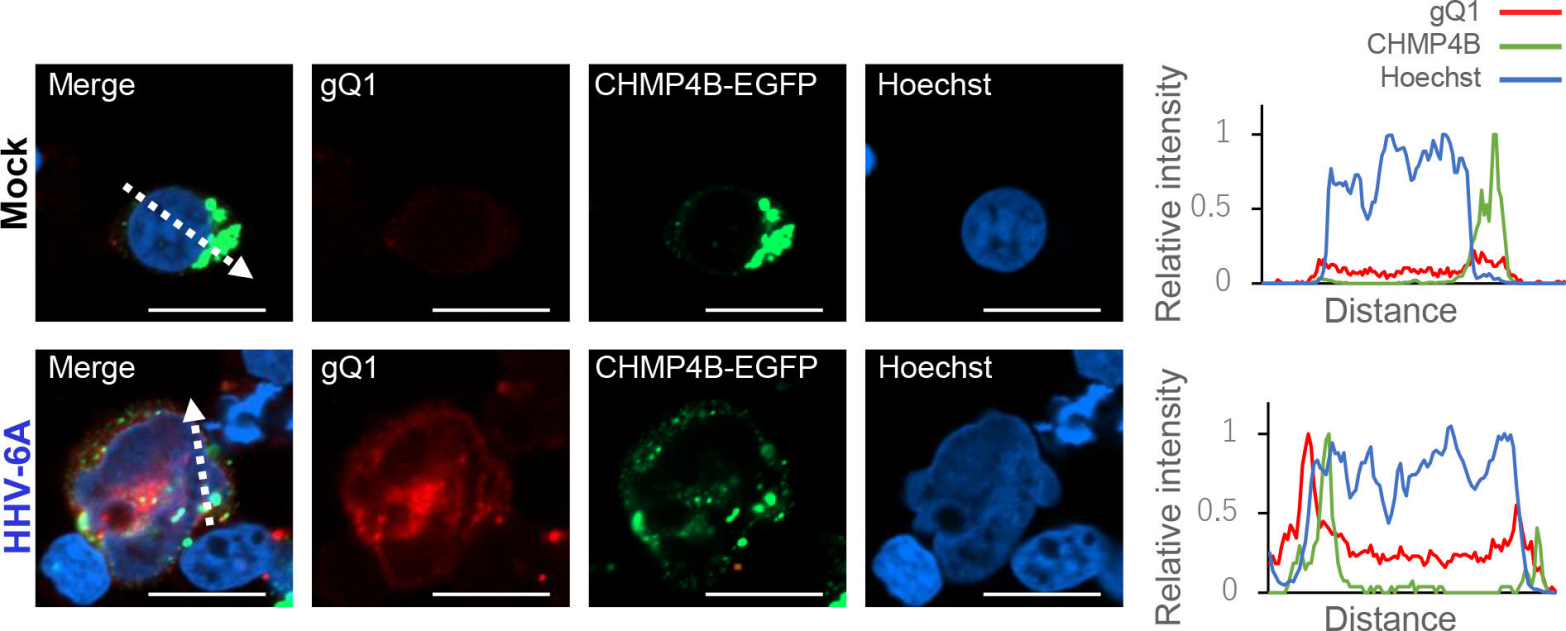
ESCRT-III protein CHMP4B accumulates at the nuclear rim and in the cytoplasm of HHV-6A-infected JJhan cells. JJhan-tetCHMP4B-EGFPneo cells were either mock-infected or infected with HHV-6A for 24 hours, followed by an additional 48 hour incubation in medium containing Dox. Then, the cells were fixed and stained with antibody against HHV-6A gQ1. Bar, 10 µm. Fluorescence intensities along the dotted lines of the images are shown to the right of each set of images.

To determine whether ESCRT-III is localized at the nuclear rim in infected cells, we constructed JJhan cells producing CHMP4B-EGFP in the presence of doxycycline (Dox). These were designated JJhan-tetCHMP4B-EGFPneo. JJhan cells were infected with HHV-6A in the presence of Dox and analyzed by immunofluorescence 72 hours later. Viral glycoprotein gQ1 was detected in about 15% of the infected cells but not in mock-infected controls (Fig. 5). In the latter, CHMP4B-EGFP was mainly detected in the cytoplasm. In the gQ1-expressing cells, CHMP4B-EGFP formed punctate structures at the nuclear rim as well as in the cytoplasm (Fig. 5). Although CHMP4B-EGFP is overexpressed in JJhan cells upon Dox induction, its expression level appears lower than that in the HeLa-CHMP4B-EGFP cells used in earlier figures, likely accounting for the relatively weak nuclear rim signal observed here. Given the difference in cellular context and expression background, the CHMP4B localization pattern is not expected to completely match that seen in HeLa cells. It is also important to note that we were unable to investigate the localization of the NEC in HHV-6A–infected cells due to the lack of available antibodies against U34 or U37. Nevertheless, it is plausible that the NEC colocalizes with CHMP4B, as CHMP4B-EGFP puncta were observed at the nuclear periphery, which is clearly delineated by Hoechst staining. These results suggest that HHV-6A infection results in the recruitment of ESCRT-III machinery, similar to what has been reported for alpha- and gammaherpesviruses (25, 26, 28).

### ESCRT-III is important for nuclear egress and replication of HHV-6A

**Fig 6:**
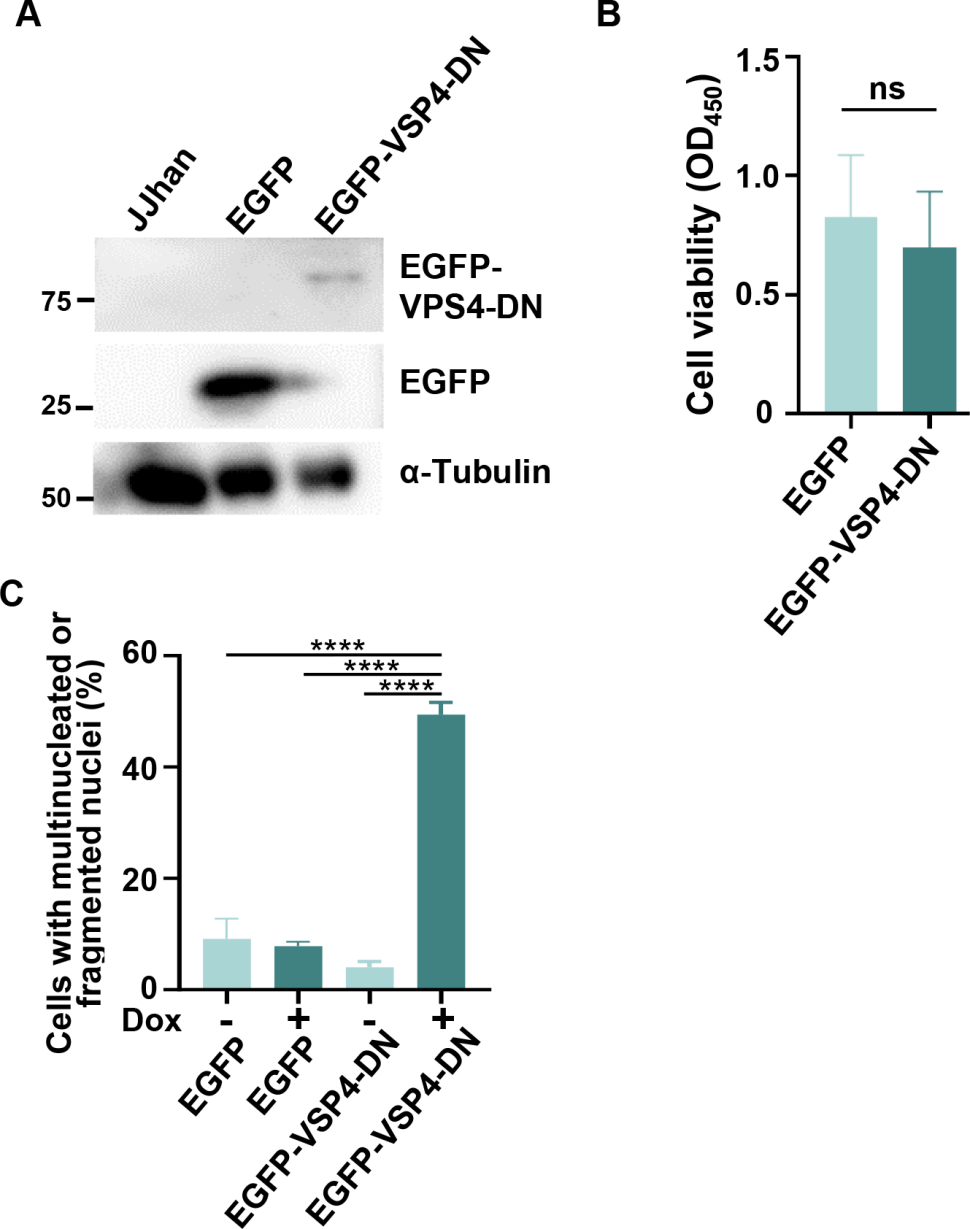
Expression of VPS4-DN in JJhan cells impairs cytokinesis. JJhan, JJhan-tetEGFP (EGFP) or JJhan-tetEGFP-VPS4-DN (EGFP-VPS4-DN) cells were incubated in medium with or without Dox for 72 hours and then analyzed by immunoblotting (**A**), cell viability assays (**B**), or immunofluorescence to detect cells with multinucleated or fragmented nuclei (**C**). Data are shown as means of three independent experiments ± standard error (ns, not significant; Student’s *t*-test for B; ****, *P* < 0.0001, Tukey’s test for C).

VPS4 AAA-ATPases disassemble ESCRT-III filaments to the monomeric state, which is essential for membrane scission and recycling of ESCRT-III proteins (20, 21). Thus, expression of a VPS4 dominant-negative allele (VPS4-DN) severely impairs ESCRT-III-mediated scission (36, 37). To investigate the role of ESCRT-III in HHV-6A replication in T cell lines, we first constructed JJhan cells producing the fusion protein of EGFP and VPS4-DN in the presence of Dox. These were designated JJhan-tetEGFP-VPS4-DN. JJhan cells expressing EGFP in the presence of Dox were also constructed as a control (JJhan-tetEGFP). These cells were treated with Dox for 72 hours, followed by immunoblotting with anti-GFP antibody. As shown in Fig. 6A, JJhan-tetEGFP or JJhan-tetEGFP-VPS4-DN cells produced EGFP or EGFP-VPS4-DN in the presence of Dox, although the expression level of EGFP-VPS4-DN was substantially lower than that of EGFP alone. This treatment had no effect on cell viability (Fig. 6B). These cells were also analyzed by immunofluorescence with Hoechst dye in the presence or absence of Dox treatment, revealing that about 50% of EGFP-VPS4-DN-expressing cells were multi-nucleated or possessed fragmented nuclei (Fig. 6C), which is a hallmark of defective ESCRT-III function (38–40).

**Fig 7:**
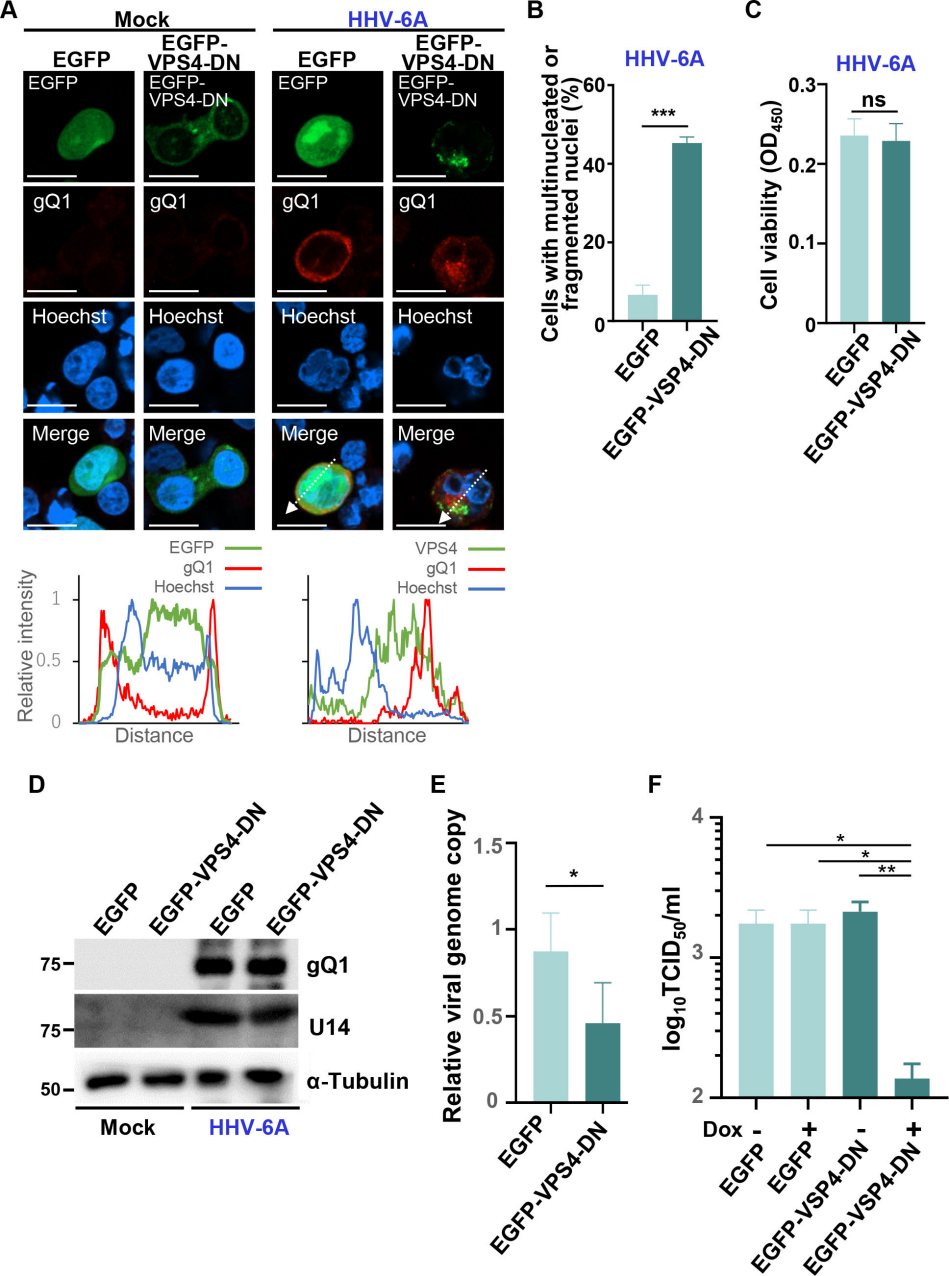
Expression of VPS4-DN in JJhan cells reduces progeny viral yields in infected cells. (**A through D**) JJhan-tetEGFP (EGFP) or JJhan-tetEGFP-VPS4-DN (EGFP-VPS4-DN) cells were infected with HHV-6A or mock-infected and incubated with medium containing Dox for 72 hours. The cells were fixed and visualized by confocal microscopy (**A**), and the number of cells with multinucleated or fragmented nuclei was quantified (**B**). The cells were also collected for cell viability assays (**C**) and immunoblot analysis (**D**). Fluorescence intensities of HHV-6A-infected JJhan-tetEGFP and JJhan-tetEGFP-VPS4-DN cells along the dotted lines of the images are shown at the bottom of each set of images. Bar, 10 µm. ***, *P* < 0.001, Student’s *t*-test. (**E**) JJhan, JJhan-tetEGFP (EGFP), or JJhan-tetEGFP-VPS4-DN (EGFP-VPS4-DN) cells were infected with HHV-6A or mock-infected and incubated with medium containing Dox. After 72 hours, viral DNA in the supernatant of the cells was collected and analyzed by qPCR. Viral genome copy numbers from JJhan-tetEGFP or JJhan-tetEGFP-VPS4-DN cells relative to JJhan cells are shown. Results are shown as means and standard errors of four independent experiments (*, *P* < 0.05, Student’s *t*-test). (**F**) JJhan-tetEGFP (EGFP) or JJhan-tetEGFP-VPS4-DN (EGFP-VPS4-DN) cells were infected with HHV-6A-BAC and incubated in medium with or without Dox. After 72 hours, the cells and the supernatant were collected, and 50% tissue culture infectious dose (TCID_50_) was determined using JJhan cells. Results are shown as means and standard errors of four independent experiments (*, *P* < 0.05; **, *P* < 0.01, Tukey’s test).

**Fig 8:**
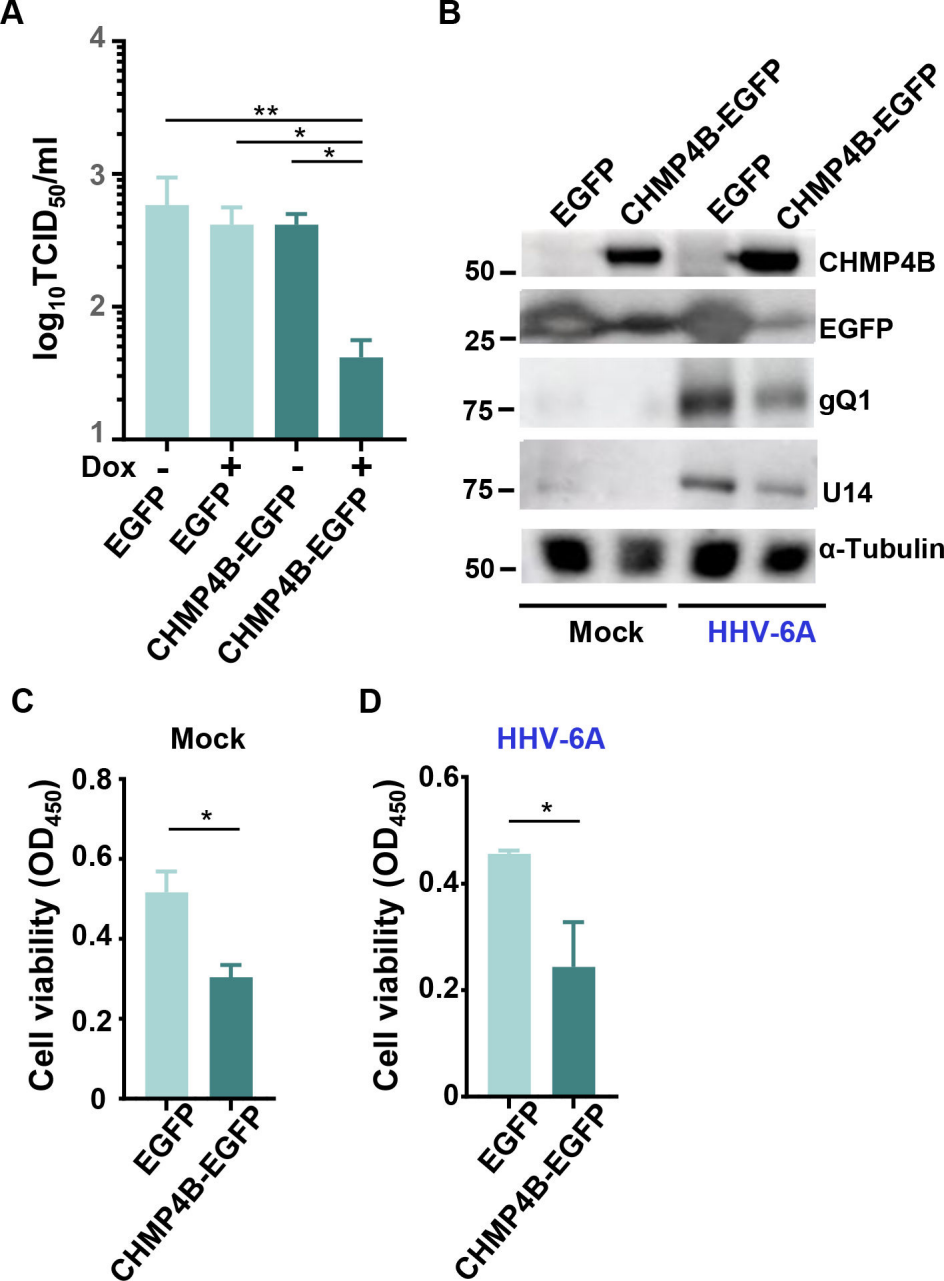
Expression of CHMP4B-EGFP in JJhan cells reduces yields of viral progeny in infected cells. (**A**) JJhan-tetEGFPneo (EGFP) or JJhan-tetCHMP4B-EGFPneo (CHMP4B-EGFP) cells were infected with HHV-6A-BAC for 24 hours, followed by further incubation in medium with or without Dox for 48 hours. The cells and supernatants were collected, and TCID_50_ was determined using JJhan cells. Results are shown as means and standard errors of four independent experiments (*, *P* < 0.05; **, *P* < 0.01, Tukey’s test). (**B through D**) JJhan-tetEGFPneo (EGFP) or JJhan-tetCHMP4B-EGFPneo (CHMP4B-EGFP) cells were mock-infected or infected with HHV-6A for 24 hours, followed by further incubation in medium with or without Dox for 48 hours. The cells were analyzed by cell viability assays (**B and C**) or immunoblotting (**D**). Data are shown as means of three independent experiments ± standard error (*, *P* < 0.05, Student’s *t*-test).

Next, these cells were infected with HHV-6A and incubated for 72 hours in the presence of Dox. As shown in Fig. 7A, EGFP-VPS4-DN induced cytoplasmic domains in which gQ1 was partially colocalized in infected JJhan-tetEGFP-VPS4-DN cells. Line intensity profile analysis (Fig. 7A, bottom) demonstrated partial overlap between EGFP-VPS4-DN and gQ1 signals at these cytoplasmic domains. These results suggest that EGFP-VPS4-DN was localized to a cytoplasmic compartment that may serve as the site of secondary envelopment, as previously reported (41, 42). These cells exhibited multinucleation or nuclear fragmentation without any reduction in viability (Fig. 7C) and produced viral protein gQ1 and U14 at similar levels to controls, suggesting that EGFP-VPS4-DN had no effect on expression of viral genes and genome replication (Fig. 7D), in contrast to what has been reported for HCMV (29). Using quantitative PCR (qPCR), we also analyzed yields of viral progeny in the supernatants of infected cells. The number of copies of the HHV-6A genome in the supernatant of infected JJhan-tetEGFP-VPS4-DN cells was moderately decreased in comparison to infected JJhan-tetEGFP cells (Fig. 7E). Furthermore, we quantified the titers of infectious viruses from these cells. JJhan-tetEGFP-VPS4-DN or JJhan-tetEGFP cells were infected with HHV-6A BAC harboring an EGFP expression cassette under the control of the HCMV IE promoter (43) in the presence or absence of Dox. At 72 hours post-infection, infected cells were collected and titrated using JJhan cells. As shown in Fig. 7F, the viral titer produced by JJhan-tetEGFP-VPS4-DN cells treated with Dox was significantly lower than that without Dox or JJhan-tetEGFP cells. Taken together, these observations suggest that ESCRT-III promotes viral replication at a stage after viral gene expression. We also examined the effect of CHMP4B-EGFP expression on viral replication, given that overexpression of ESCRT-III components can impair ESCRT-III functions similar to VPS4-DN (44). As shown in Fig. 8A, viral titers 72 hours post-infection were lower in JJhan-tetCHMP4B-EGFPneo than in the control JJhan-tetEGFPneo cells or the same cells cultured without Dox. Notably, CHMP4B-EGFP expression led to a reduction in the accumulation of viral proteins and exhibited a degree of cytotoxicity (Fig. 8B through D).

**Fig 9:**
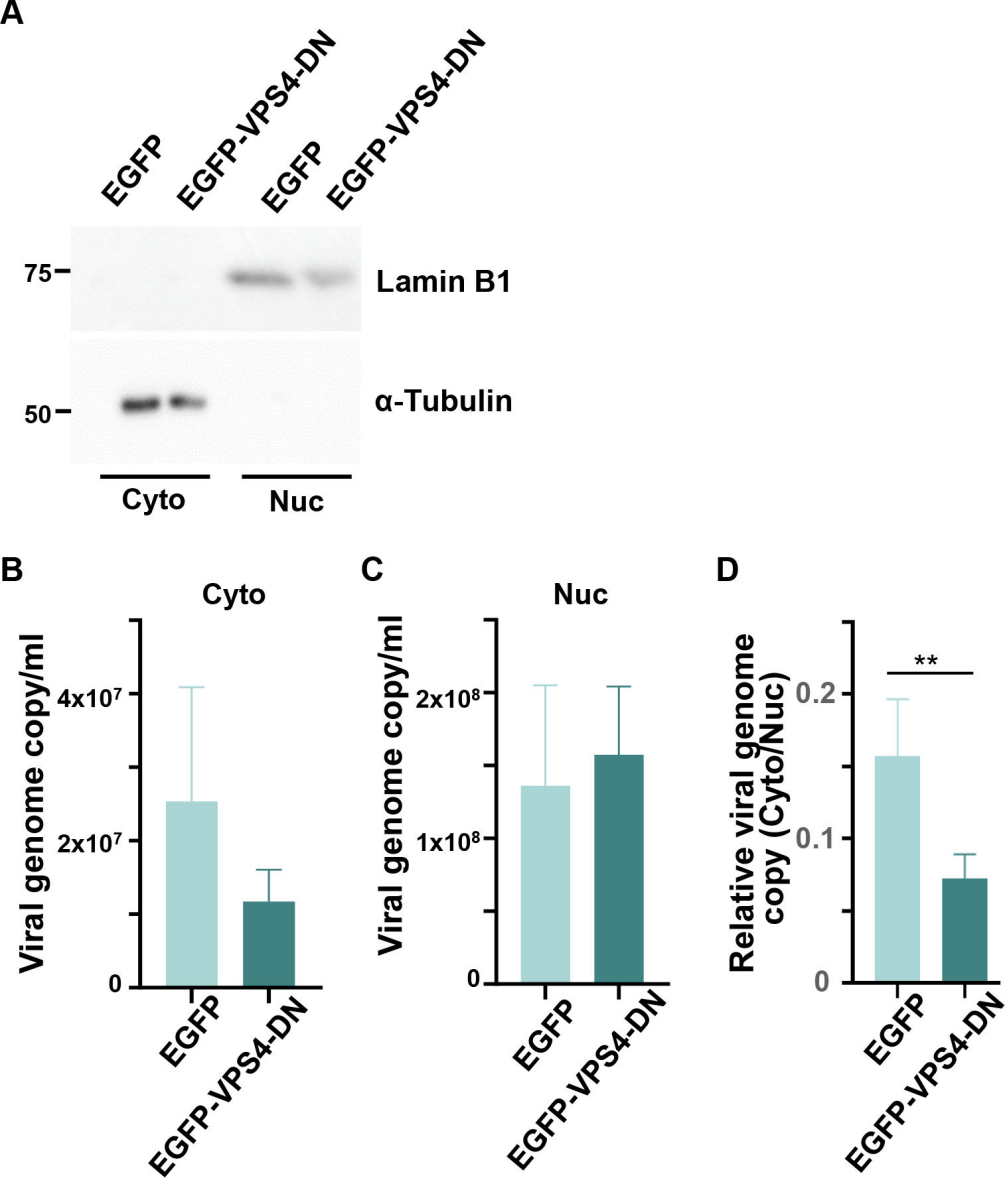
Expression of VPS4-DN in JJhan cells impairs the nucleo-cytoplasmic transport of HHV-6A capsids. (**A**) Nuclear (Nuc) and cytoplasmic (Cyto) fractions of Jhan-tetEGFP (EGFP) or JJhan-tetEGFP-VPS4-DN (EGFP-VPS4-DN) cells were separated and analyzed by immunoblotting using antibodies against α-tubulin (cytoplasmic marker) and lamin B1 (nuclear marker). (**B through D**) JJhan-tetEGFP or JJhan-tetEGFP-VPS4-DN cells were infected with HHV-6A. After 72 hours, nuclear (**B**) and cytoplasmic fractions (**C**) were separated, followed by quantification of viral genome copy numbers by qPCR. (**D**) The ratios of nuclear to cytoplasmic viral genome copies are shown. Results are means and standard errors of four independent experiments (**, *P* < 0.01, Student’s *t*-test).

Next, we analyzed the role of ESCRT-III in HHV-6A capsid nuclear egress. To determine the efficacy of this process, we performed subcellular fractionation experiments. In both JJhan-tetEGFP and JJhan-tetEGFP-VPS4-DN cells, α-tubulin and lamin B1 were detected only in the cytoplasmic and nuclear fractions, respectively (Fig. 9A), suggesting that fractionation of nucleus and cytoplasm had been appropriately accomplished. Next, JJhan-tetEGFP or JJhan-tetEGFP-VPS4-DN cells were infected with HHV-6A for 72 hours in the presence of Dox, followed by subcellular fractionation. Viral genomes were collected from both fractions and quantified by qPCR. As shown in Fig. 9B and C, the cytoplasmic viral genome copy number tended to be reduced in EGFP-VPS4-DN-expressing cells, although the difference was not statistically significant. Notably, the cytoplasmic-to-nuclear ratio of viral genome copies was modestly decreased in these cells (Fig. 9D). These observations suggested that ESCRT-III is important for nucleocytoplasmic transport of HHV-6A nucleocapsids.

### ESCRT-III adaptor ALIX is important for HHV-6A replication

**Fig 10:**
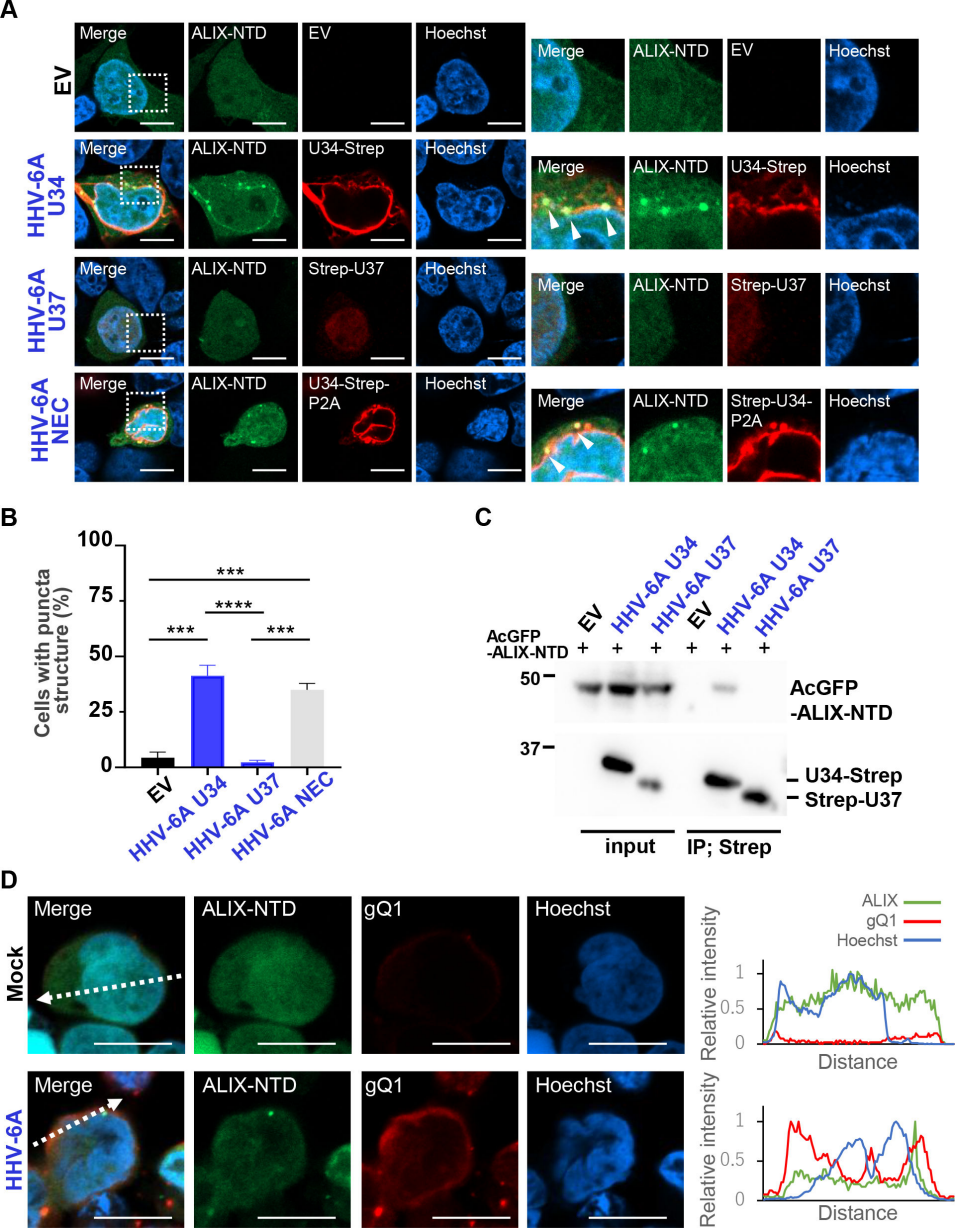
ESCRT-III adaptor ALIX interacts with HHV-6A U34 and localizes at the nuclear rim in infected cells. (**A and B**) HEK293T cells were transfected with empty vector (EV) or plasmids HHV-6A U34-Strep, Strep-HHV-6A U37, or HHV-6A U34-Strep/Flag-U37 coupled with AcGFP-ALIX-NTD plasmid. These cells were analyzed at 48 hours after transfection by immunofluorescence (**A**), and the percentage of cells displaying punctate structures of AcGFP-ALIX-NTD was determined by counting 100 cells (**B**). Each image in the right-hand panels is the magnified image of the boxed area in the left-hand panels. Results are means and standard errors of three independent experiments (***, *P* < 0.001; ****, *P* < 0.0001, Tukey’s test). Arrowheads indicate the accumulation of ALIX-NTD. (**C**) The cells transfected as described in (**A**) were lysed, and the extracts were precipitated using Strep-Tactin beads, followed by immunoblotting. (**D**) JJhan-tetAcGFP-ALIX-NTDneo cells were either mock-infected or infected with HHV-6A. After 72 hours, the cells were fixed, stained with an antibody against HHV-6A gQ1. Bar, 10 µm. Fluorescence intensities along the dotted lines of the images are shown to the right of each set of images. EV, empty vector. Bar, 10 µm.

The ESCRT-III adaptor ALIX is reported to be important for the recruitment of ESCRT-III to nuclear membranes through HSV-1 UL34 or EBV BFRF1 (25, 26, 28), both of which are homologs of HHV-6A U34. In particular, the ALIX N-terminal domain (NTD) has been reported to interact with both HSV-1 UL34 and EBV BFRF1. To address whether ALIX interacts with HHV-6A U34, we performed three series of experiments. First, HEK293T cells transfected with plasmids to express HHV-6A U34-Strep, Strep-HHV-6A U37, U34-Strep/Flag-U37, or empty plasmid coupled with AcGFP-ALIX-NTD were analyzed by immunofluorescence. ALIX-NTD was distributed diffusely in the nucleus and cytoplasm in the cells transfected with empty plasmid, but HHV-6A U34 expression redistributed ALIX-NTD to punctate structures in the cytoplasm (Fig. 10A and B). However, HHV-6A U37 expression did not result in any punctate ALIX-NTD structures. Notably, co-expression of HHV-6A U34 and U37 led to the redistribution of ALIX-NTD punctate structures at the nuclear rim. In a second experiment, we performed co-precipitation of HEK293T cells transfected with HHV-6A U34-Strep, Strep-HHV-6A U37, or empty plasmid coupled with AcGFP-ALIX-NTD. These cells were then lysed and precipitated with Strep Tactin beads. As shown in Fig. 10C, AcGFP-ALIX-NTD was not precipitated from the lysates of the cells transfected with Strep-HHV-6A U37 expression plasmid or empty plasmid. In contrast, AcGFP-ALIX-NTD was precipitated from lysates of cells expressing Strep-HHV-6A U34. Third and last, ALIX-NTD was localized in the nucleus and formed punctate structures at the nuclear rim of HHV-6A-infected cells (Fig. 10D). Taken together, these results suggest that HHV-6A U34 specifically interacts with ALIX, similar to HSV-1 UL34 and EBV BFRF1 (25, 26, 28).

**Fig 11:**
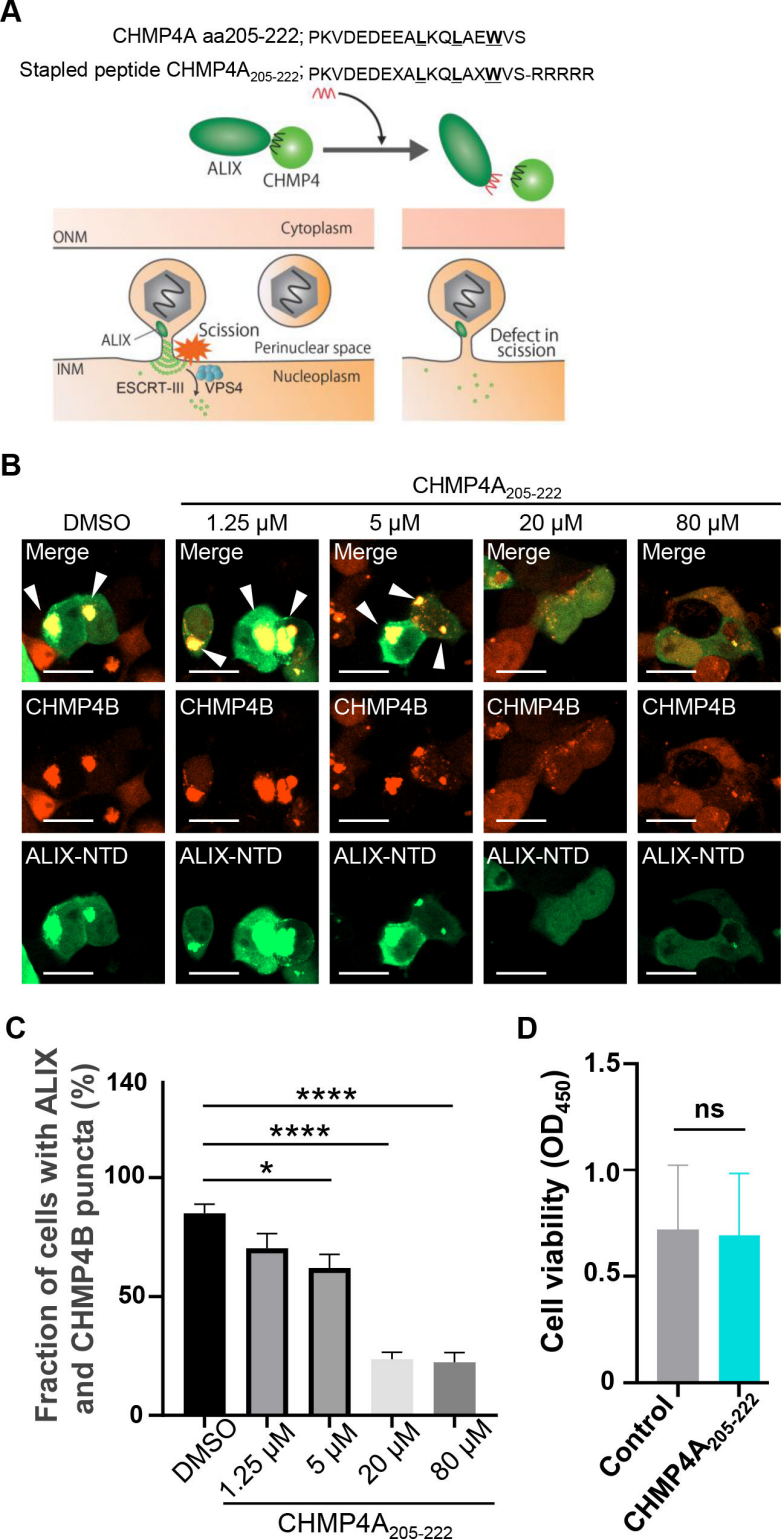
The stapled peptide CHMP4A_205-222_ blocks ALIX and CHMP4B interactions. (**A**) Schematic diagram of stapled peptide CHMP4A_205-222_, designed to impair ALIX-mediated ESCRT-III function. (**B and C**) HEK293T cells were transfected with plasmids for AcGFP-ALIX-NTD and TagRFP-CHMP4B and incubated in the indicated concentrations of the CHMP4A_205-222_ peptide. After 48 hours, the cells were analyzed by confocal microscopy (**B**). Arrowheads indicate the accumulation of CHMP4B and ALIX-NTD. Bar, 50 µm. Percentages of cells displaying punctate structures of AcGFP-ALIX-NTD and TagRFP-CHMP4B were estimated by counting 100 cells (**C**). (**D**) JJhan cells were treated with 20 µM of CHMP4A_205-222_ peptide or DMSO for 72 hours, followed by cell viability assays. Data are shown as means of three independent experiments ± standard error (*, *P* < 0.05; ****, *P* < 0.0001, Tukey’s test for C; ns, not significant; Student’s *t*-test for D).

**Fig 12:**
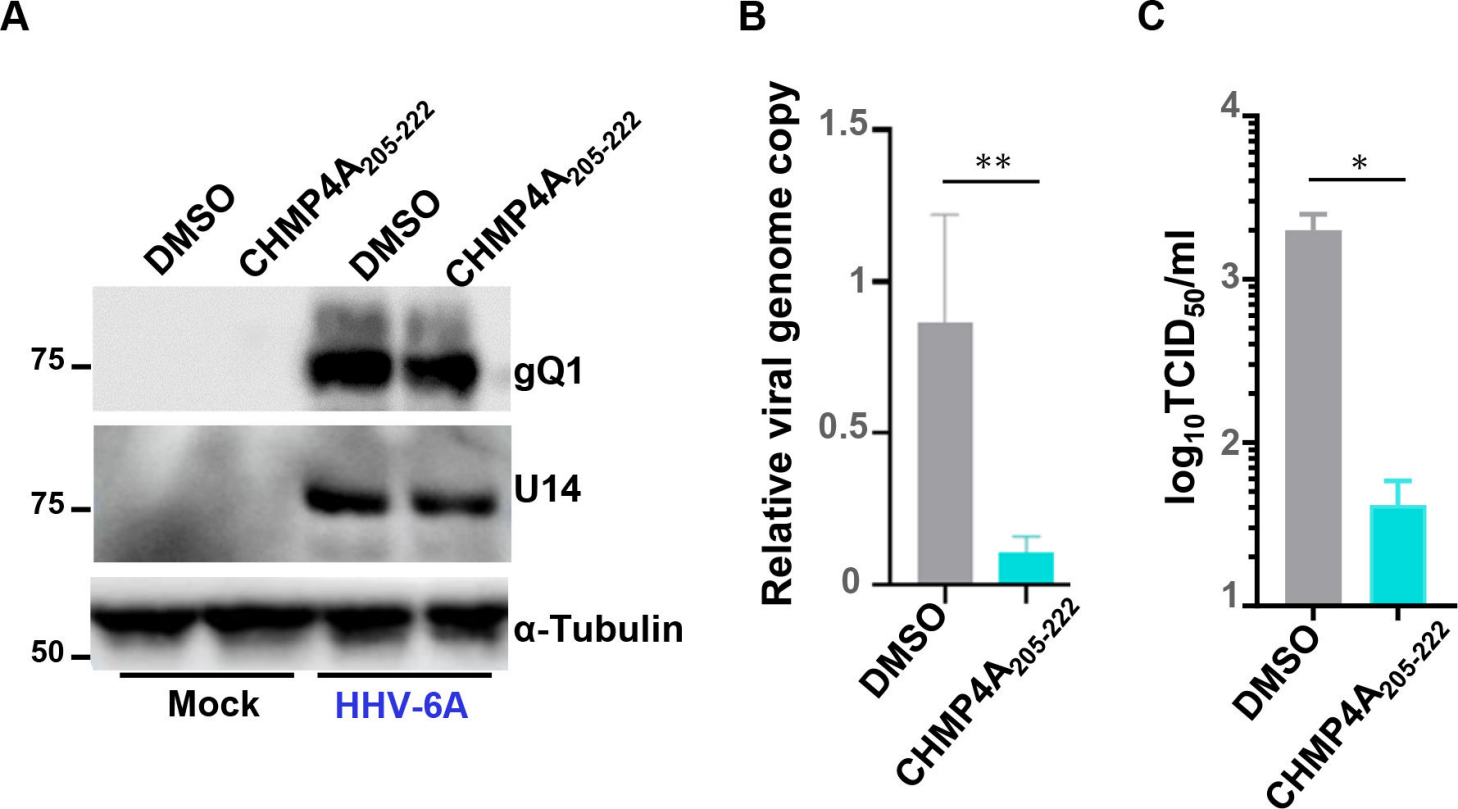
The stapled peptide CHMP4A_205-222_ impairs HHV-6A replication. (**A**) JJhan cells were mock-infected or infected with HHV-6A and incubated with medium containing DMSO or 20 µM of the stapled peptide CHMP4A_205-222_. After 72 hours, the cells were collected and analyzed by immunoblotting. (**B**) JJhan cells were mock-infected or infected with HHV-6A and incubated with medium containing DMSO or 20 µM of the stapled peptide CHMP4A_205-222_ or medium without DMSO. After 72 hours, viral DNA in the supernatant of the cells was collected and analyzed by qPCR. Viral genome copy numbers relative to cells incubated without DMSO are shown. Results are means and standard errors of four independent experiments (**, *P* < 0.01, Student’s *t*-test). (**C**) JJhan cells were infected with HHV-6A-BAC in the presence or absence of 20 µM of the stapled peptide CHMP4A_205-222_ for 72 hours. Cells and supernatants were subjected to freeze–thaw cycles, and TCID_50_ was determined using JJhan cells. Results are shown as means and standard errors of four independent experiments (*, *P* < 0.05, Student’s *t*-test).

We next investigated the role of ALIX in HHV-6A replication. As ALIX is essential for cytokinesis (38, 39), we aimed to transiently inhibit ALIX and ESCRT-III interactions. It has been reported that ALIX NTD binds ESCRT-III component CHMP4 and that this is crucial for ALIX-dependent ESCRT-III function (45). Solved crystal structures have revealed that CHMP4A residues 205-222 form an amphipathic helix that binds across the concave surface of ALIX-NTD (45). Stapled peptides are peptides locked into their bioactive alpha-helical conformation through site-specific introduction of a chemical brace, an all-hydrocarbon staple to enhance stability and cell permeability (46). Accordingly, we synthesized stapled peptides harboring CHMP4A_205-222_ to inhibit the interaction between ALIX and CHMP4 proteins (Fig. 11A). Using live imaging, we first analyzed whether the CHMP4A_205-222_ peptide inhibits ALIX and CHMP4 interactions. HEK293T cells were transfected with a plasmid to express AcGFP-ALIX-NTD and TagRFP-CHMP4B in the presence or absence of the CHMP4A_205-222_ peptide. As expected, AcGFP-ALIX-NTD and TagRFP-CHMP4B formed punctate structures in the cytoplasm (Fig. 11B), but CHMP4A_205-222_ peptide significantly reduced the fraction of cells exhibiting such structures in a dose-dependent manner (Fig. 11C), suggesting that this peptide impairs ALIX-mediated ESCRT-III function. It is important to note that we did not employ a control peptide in this experiment; instead, we equalized the amount of dimethyl sulfoxide (DMSO), the solvent used for peptide delivery, across all conditions. Therefore, the observed effect of the stapled peptide may potentially result from non-specific interactions independent of its sequence. JJhan cells treated with CHMP4A_205-222_ peptide for 72 hours did not show any cytotoxicity (Fig. 11D), indicating that the peptide does not compromise cell viability under these conditions. JJhan cells infected with HHV-6A were then treated or mock-treated with the CHMP4A_205-222_ peptide for 72 hours. Viral genomes were then collected from the supernatants for quantification. In a separate experiment, JJhan cells were infected with HHV-6A BAC, in the presence or absence of the CHMP4A_205-222_ peptide. After 72 hours, both the cells and supernatants were subjected to freeze–thaw cycles, and the resulting lysates were used to infect fresh JJhan cells to assess viral infectivity, including both cell-associated and released virions. As shown in Fig. 12A, the same amount of viral gQ1 and U14 was produced in the presence or absence of the CHMP4A_205-222_ peptide. However, both the quantity of progeny viral genomes and the infectious viral titers were reduced in the presence of the CHMP4A_205-222_ peptide (Fig. 12B and C). These results suggest that the ESCRT-III adaptor ALIX promotes HHV-6A replication.

**Fig 13:**
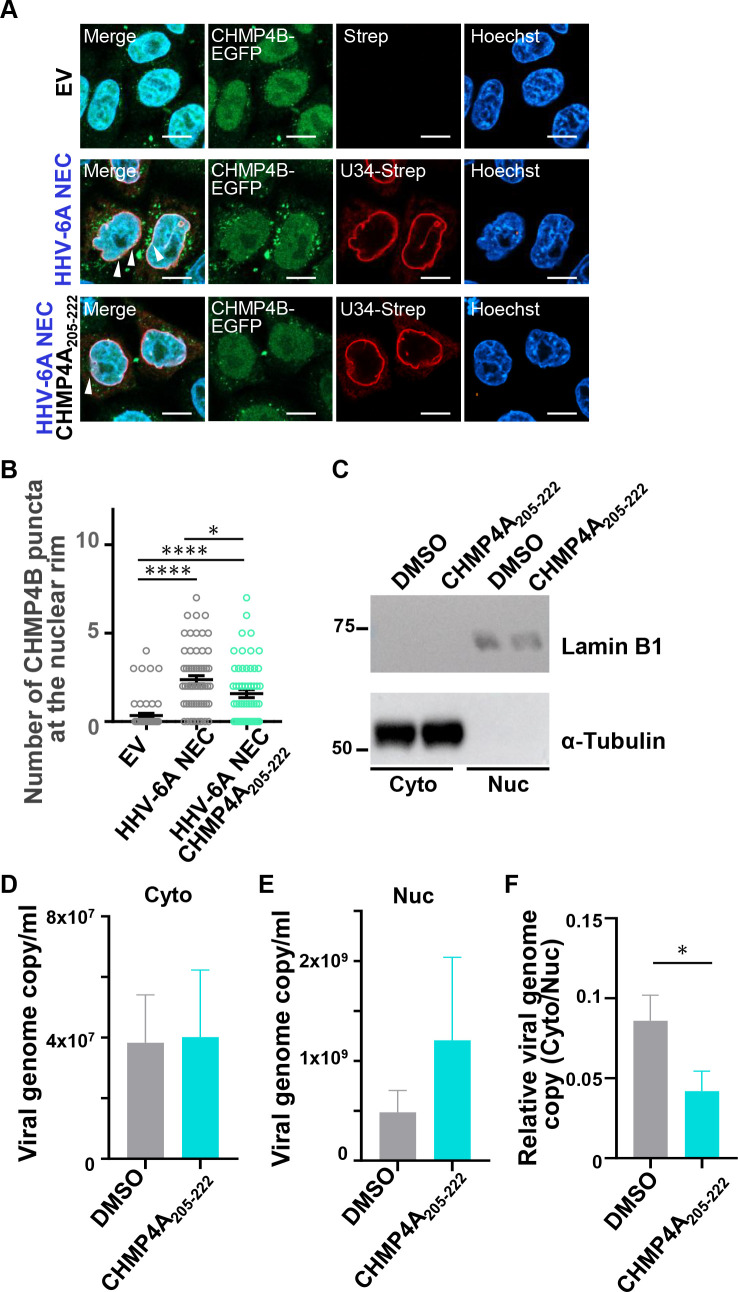
The stapled peptide CHMP4A_205-222_ impairs nucleo-cytoplasmic transport of HHV-6A capsids. (**A**) HeLa-CHMP4B-EGFP cells were transfected with plasmids encoding HHV-6A U34-Strep/Flag-U37, followed by incubation in medium containing 20 µM of the stapled peptide CHMP4A_205-222_. After 48 hours, the cells were analyzed by immunofluorescence with the indicated antibodies. Arrowheads indicate the accumulation of CHMP4B. EV, empty vector. Bar, 10 µm. The CHMP4B-EGFP-positive punctate structures at the nuclear rim in the experiment in (**A**) were quantified. Data are shown as the mean ± standard errors for 60 cells and are representative of three independent experiments (*, *P* < 0.05; ****, *P* < 0.0001, Tukey’s test). (**C**) Nuclear (Nuc) and cytoplasmic (Cyto) fractions of Jhan cells treated with 20 µM of the stapled peptide CHMP4A_205-222_ for 72 hours were separated and analyzed by immunoblotting using antibodies against lamin B1 (nuclear marker) and α-tubulin (cytoplasmic marker). (**D through F**) JJhan cells were mock-infected or infected with HHV-6A and incubated with medium containing DMSO or 20 µM of the stapled peptide CHMP4A_205-222_. After 72 hours, nuclear (**D**) and cytoplasmic fractions (**E**) were separated, followed by quantification of viral genome copy numbers by qPCR. (**F**) The ratios of nuclear to cytoplasmic viral genome copies are shown. Results are means and standard errors of four independent experiments (*, *P* < 0.05, Student’s *t*-test).

**Fig 14:**
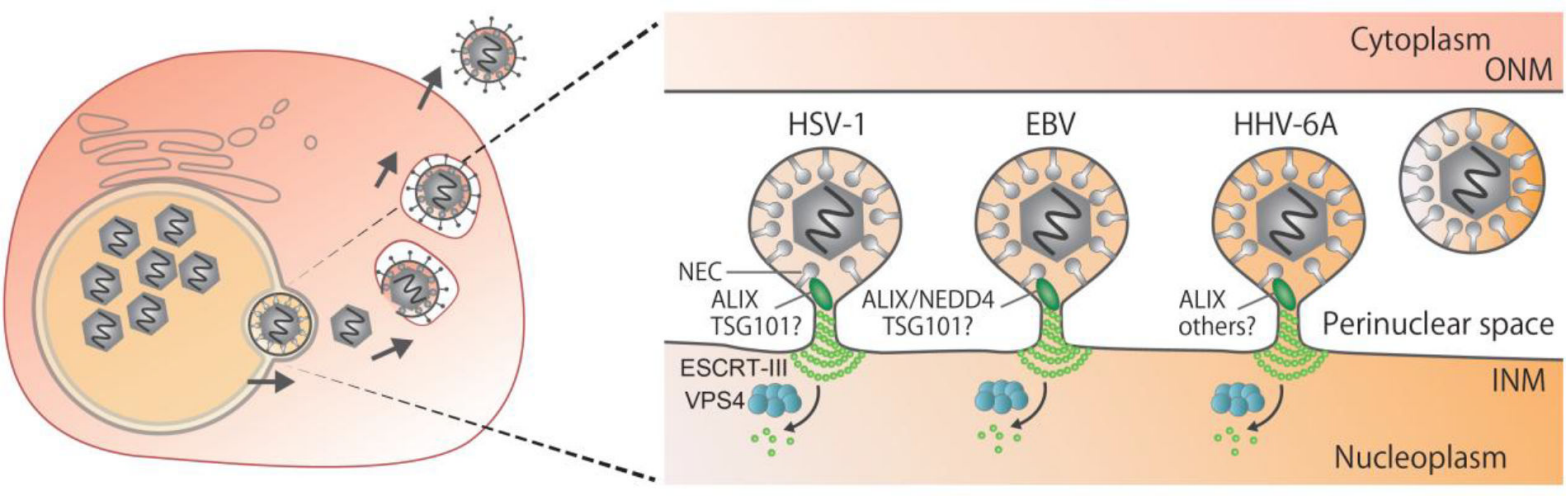
Schematic illustration of nuclear egress of herpesviruses. After genome replication in the nucleus, viral capsids in the nucleus bud through the INM to form vesicles in the perinuclear space that fuse with the ONM for release into the cytoplasm. During budding at the INM, the NEC mediates scission by recruitment of ESCRT-III via ALIX or NEDD4.

Finally, we investigated whether ALIX contributes to the nuclear egress of HHV-6A. To address the role of ALIX in CHMP4B recruitment by the HHV-6A NEC, HeLa-CHMP4B-EGFP cells were transfected with an HHV-6A NEC expression plasmid in the presence or absence of the CHMP4A_205-222_ peptide. Consistent with the results shown in Fig. 3B, CHMP4B-EGFP formed punctate structures colocalizing with U34-Strep in the absence of the CHMP4A_205-222_ peptide. In contrast, treatment with the CHMP4A_205-222_ peptide reduced the number of CHMP4B-EGFP puncta at the nuclear rim (Fig. 13A and B). We next examined the role of ALIX in HHV-6A capsid nuclear egress. JJhan cells were incubated with or without the CHMP4A_205-222_ peptide, followed by subcellular fractionation. In both peptide-treated and untreated cells, α-tubulin and lamin B1 were detected exclusively in the cytoplasmic and nuclear fractions, respectively (Fig. 13C), indicating successful separation of nuclear and cytoplasmic components. Subsequently, JJhan cells were infected with HHV-6A for 72 hours in the presence or absence of the CHMP4A_205-222_ peptide and then subjected to subcellular fractionation. Viral genomes were extracted from both fractions and quantified by qPCR. While the absolute copy number of viral genomes in the cytoplasmic fraction was not reduced upon CHMP4A_205-222_ treatment (Fig. 13D and E), the cytoplasmic-to-nuclear ratio of viral genome copy number was decreased in peptide-treated cells (Fig. 13F). Although this difference reached statistical significance at *P* = 0.05, the relatively large variability in viral genome quantification and the pronounced difference in genome abundance between nuclear and cytoplasmic fractions limit the strength of this conclusion, and we therefore interpret this finding as a trend rather than a definitive effect. Nevertheless, these results suggest that the ESCRT-III adaptor ALIX interacts with HHV-6A NEC to facilitate efficient nuclear egress of viral capsids and promote viral replication, potentially in cooperation with other adaptor proteins (Fig. 14).

## DISCUSSION

Virion structures and mechanisms of their formation are widely conserved in *Herpesviridae* (47). However, although ESCRT-III is crucial for nuclear egress and secondary envelopment in alpha- and gammaherpesviruses (48), it has recently been reported that it is not required for replication of the betaherpesvirus HCMV (29). Here, we show that exogenous expression of the NEC from HHV-6A or HCMV resulted in the recruitment of ESCRT-III to the nuclear rim in a similar manner to what is observed in alpha- and gammaherpesviruses (22–24). Consistent with this, ESCRT-III was detected at the nuclear rim in HHV-6A-infected cells. Notably, overexpression of CHMP4B-EGFP can sometimes exert dominant-negative effects on ESCRT-III function. Therefore, the possibility that the recruitment of CHMP4B to the nuclear rim observed in our experiments is non-functional cannot be entirely excluded. Nevertheless, inhibition of this pathway, either by expression of VPS4-DN or treatment with the peptide that blocks the ALIX-CHMP4 interaction, led to modestly reduced production of viral progeny and impaired nucleocytoplasmic transport of viral DNA, highlighting the important role of ESCRT-III in nuclear egress and replication of HHV-6A.

ESCRT-III is the sole cellular system responsible for reverse-topology scission and is exploited by many viruses for the final stage of viral budding (49). Some viruses, such as the influenza virus, can replicate independently of ESCRT-III by mediating scission with their own proteins (50). Similar to the influenza M2 protein, purified HSV-1 NEC was reported to cleave giant unilamellar vesicles *in vitro* (11). Thus, nuclear egress of herpesviruses may not necessarily require ESCRT-III activity. However, there is currently no evidence that NEC cleaves the nuclear membrane in cells without the aid of host factors. Our results revealed that NEC of all three subfamilies in *Herpesviridae* have the ability to recruit ESCRT-III protein to the nuclear rim, suggesting a common contribution of this mechanism to nuclear egress. It is currently unclear why inhibition of ESCRT-III reportedly fails to inhibit HCMV. Possibly both ESCRT-III as well as viral proteins mediate membrane scission redundantly, such that the contribution of ESCRT-III is not crucial for HCMV nuclear egress under some conditions.

The interspecies interaction of NEC components was analyzed in detail previously in HCMV and murine cytomegalovirus (MCMV) (34). Interactions between HCMV UL50 and MCMV M53 (UL53 homolog), as well as MCMV M50 (UL50 homolog) and HCMV UL53, were documented by co-immunoprecipitation and immunofluorescence, whereas no interactions with alpha- or gammaherpesvirus homologs were detected (34). Furthermore, recombinant MCMV harboring HMCV UL50 instead of M50 replicates to the same extent as wild-type MCMV (34). Similarly, we have shown that the NEC components of HCMV and HHV-6A form a complex at the nuclear rim. Thus, the NEC components from the same subfamily may be interchangeable, and their function is highly conserved. Several host factors are known to interact with HCMV NEC. For example, the viral protein kinase UL97 and cellular protein p32 associate with HCMV NEC and increase lamin phosphorylation to disrupt the lamina meshwork (3). These features are possibly conserved in HHV-6A NEC.

ESCRT-III has an important role not only in virion formation but also in cell division, nuclear membrane maintenance, and resistance to cell death. The present results using VPS4-DN or peptide do not exclude an indirect effect on the broad function of ESCRT-III. Notably, in the case of HCMV, earlier reports indicated that inhibition of the ESCRT machinery does not prevent viral particle formation, but may instead impair viral replication through indirect mechanisms (29). Since we found an interaction between ALIX and HHV-6A NEC, the importance of ESCRT-III in nuclear egress may be analyzed more precisely in the future by inhibiting these protein-protein interactions, coupled with electron microscopic analysis. A limitation of the present study is that we did not address whether ESCRT-III contributes to secondary envelopment of HHV-6A. VPS4-DN has been shown to co-localize with several viral factors in the HCMV and HHV-6A assembly compartment (29, 30, 41) (Fig. 7A).

Three ESCRT-III adaptors are known to mediate budding of different viruses from many families (51, 52). These ESCRT-associated proteins facilitate virus egress through conjugation with characteristic motifs designated late or L-domains (51, 52). Briefly, the ESCRT-I protein TSG101 binds the PTAP motif, and ALIX binds the YPXL motif, whereas proteins of the NEDD4 ubiquitin ligase family bind the PPXY motif (51, 52). We have shown that the NEC component HHV-6A U34 interacts with ALIX, despite L-domain motifs not being present in the HHV-6 U34 sequence. It has been reported that the HSV-1 NEC interacts with ALIX, whereas the EBV NEC interacts with ALIX and NEDD4 family proteins (25–28). In addition, compounds targeting the N-terminal domain of the ESCRT-I protein TSG101 impair HSV-1 and EBV nuclear egress, suggesting a potential role of TSG101 in nuclear egress (53, 54). In the present study, we showed that inhibition of ALIX reduced HHV-6A replication but did not completely block it. Thus, multiple adaptors in addition to ALIX may promote nuclear egress and/or secondary envelopment of HHV-6A.

## MATERIALS AND METHODS

### Cells and viruses

HEK293T cells were cultured in Dulbecco’s modified Eagle medium (DMEM) supplemented with 8% fetal bovine serum (FBS) (26, 55). HeLa-CHMP4B-EGFP and Plat-GP cells were cultured in DMEM supplemented with 10% FBS, and 1 µg/mL puromycin was additionally added for the selection of HeLa-CHMP4B-EGFP cells (26). The human T-lymphoblastoid cell line JJhan was cultured in RPMI-1640 medium containing 8% FBS (56). Umbilical cord blood mononuclear cells (CBMCs) were cultured as described previously (57). CBMCs were purchased from the Cell Bank of the RIKEN BioResource Center, Tsukuba, Japan, and used to make viral stocks. The HHV-6A strain U1102 was used for this study, and HHV-6A cell-free virus was prepared from CBMCs as described previously (57). Briefly, CBMCs infected with HHV-6A U1102 were lysed by freezing and thawing once at −80°C. Cell debris was removed by centrifugation at 1,500 × *g* for 5 minutes, and the supernatants were then used for virus infections. For infecting the target cell line JJhan with HHV-6A virus stock, 2 × 10^5^ cells were collected by centrifugation, resuspended in 1.5 mL of RPMI medium with 200 µL of virus stock containing 1 × 10^7^ genome copies of the virus or medium only as a mock control. The cells were then centrifuged at 35°C for 30 minutes at 1,700 × *g* in 24-well plates, followed by culturing in RPMI supplemented with 2% FBS. The recombinant virus HHV-6A BAC, which contains the genome of the HHV-6A strain U1102 along with a BAC sequence and an EGFP expression cassette driven by the HCMV IE1 promoter inserted between the U53 and U54 loci (43), was also used in this study. The virus was propagated and handled using the same procedures as described for the HHV-6A U1102 strain. Transfection experiments were performed using Lipofectamine 3000 (Thermo Fisher Scientific).

### Titration of infectivity

A total of 1 × 10⁵ JJhan cells was collected by centrifugation and resuspended in 500 µL of RPMI medium. Subsequently, 100 µL of virus stock containing 2 × 10^6^ genome copies was added to the cell suspension. The mixture was transferred to a 24-well plate and centrifuged at 1,700 × *g* for 30 minutes at 35°C to enhance viral adsorption. After centrifugation, the cells were cultured in RPMI medium with 2% FBS. Three days post-infection, the cells were subjected to a freeze–thaw cycle to release progeny virus. The harvested supernatant was serially diluted and incubated with 1 × 10^4^ JJhan cells in 96-well plates. Four days later, infectivity was assessed based on fluorescence in infected cells. The 50% tissue culture infectious dose (TCID_50_) was calculated using the Spearman–Kärber method.

### Plasmids

HHV-6A U34-Strep, Flag-HHV-6A U37, Strep-HHV-6A U37, HCMV UL50-Strep, and Flag-HCMV UL53 plasmids were described previously (8). The HHV-6A U34-Strep/Flag-HHV-6A U37 and HCMV UL50-Strep/Flag-HCMV UL53 co-expression plasmid encodes the bipartite fusion protein of HHV-6A U34-Strep or HCMV UL50-Strep, P2A self-cleaving peptides, and Flag-HHV-6A U37 or Flag-HCMV UL53 to produce U34-Strep-P2A and Flag-U37 or UL50-Strep-P2A and Flag-UL53 separately (8).

Plasmid pPIDCB (Addgene plasmid # 169899) is an empty Dox-inducible vector with piggyBac (58). The coding sequence of mScarlet-1 in pPIDCB was replaced with that of EGFP or EGFP-VPS4-DN amplified from pEGFP-VPS4A-EQ (44) (kindly provided by W. I. Sundquist) to construct Dox-inducible EGFP or EGFP-VPS4-DN vectors with piggyBac designated pPIDCB-EGFP or pPIDBC-EGFP-VPS4-DN, respectively. Plasmid pTagRFP-CHMP4B encoding a fusion protein of TagRFP and CHMP4B (TagRFP-CHMP4B) was constructed by cloning CHMP4B cDNA, amplified by PCR from pCMV(Δ5)-CHMP4B (59) (kindly provided by W. I. Sundquist), into pTagRFP-N1 (35). Plasmid pAcGFP-ALIX-NTD encoding a fusion protein of AcGFP and ALIX-NTD (AcGFP-ALIX-NTD) was constructed by cloning ALIX cDNA amino acid 1-358, amplified by PCR from pCI-FLAG-ALIX (60) (kindly provided by W. I. Sundquist), into pAcGFP-C1 (Clontech).

The coding sequence of mClover3 in pPIDCB was replaced with that of the Neomycin resistance gene amplified from pcDNA3.1 (Thermo Fisher Scientific) to construct pPIDCBneo. The coding sequence of mScarlet-1 in pPIDCBneo was replaced with that of EGFP, CHMP4B-EGFP, or AcGFP-ALIX-NTD from pEGFP-N1 (Clontech), pCHMP4B-EGFP (26), or pAcGFP-ALIX-NTD and designated pPIDCBneo-EGFP, pPIDBCneo-CHMP4B-EGFP, or pPIDBCneo-AcGFP-ALIX-NTD, respectively.

pCHMP6-EGFP encoding a fusion protein of CHMP6 and EGFP was constructed by cloning CHMP6 cDNA, amplified by PCR from pGEX-CHMP6 (kindly provided by W. I. Sundquist), into pEGFP-N1 in-frame with EGFP. DNA fragments of pCHMP6-EGFP encoding CHMP6-EGFP were cloned into pMxs-puro to construct pMxs-CHMP6-EGFP-puro.

### Construction of cells

JJhan cells were transfected with pPIDCB-EGFP or pPIDBC-EGFP-VPS4-DN coupled with the hyPBase expression cassette amplified by PCR from pPB[Exp]-Puro-CAG>hyPBase (Vector Builder), using the NEPA Super Electroporator (NEPAGENE) following the manufacturer’s instructions. GFP-positive cells were then sorted by On-chip Sort (On-chip Biotechnologies, Tokyo) and designated JJhan-tetEGFP and JJhan-tetEGFP-VPS4-DN cells, respectively. For induction of EGFP or EGFP-VPS4-DN, these cells were treated with 1 µg/mL Dox for 72 hours.

JJhan cells were transfected with pPIDCBneo-EGFP, pPIDBCneo-CHMP4B-EGFP, or pPIDBCneo-AcGFP-ALIX-NTD coupled with the hyPBase expression cassette described above. These cells were cultured with RPMI medium containing 500 µg/mL G418 to construct JJhan-tetEGFPneo, JJhan-tetCHMP4B-EGFPneo, and JJhan-tetAcGFP-ALIX-NTDneo cells, respectively. For induction of EGFP, CHMP4B-EGFP, or AcGFP-ALIX-NTD, these cells were treated with 1 µg/mL Dox for 48 hours.

Plat-GP cells, a 293T-derived murine leukemia virus-based packaging cell line, were co-transfected with pMxs-CHMP4B-EGFP-puro and pMDG encoding vesicular stomatitis virus envelope protein G (26). Supernatants were harvested 48 hours post-transfection. HeLa cells were then transduced with the retrovirus-containing supernatants of the transfected Plat-GP cells and selected with 1 µg puromycin**/**mL. Resistant cells transduced by recombinant retrovirus derived from pMxs vectors were cloned from single colonies and designated HeLa-CHMP6-EGFP.

### Antibodies

For immunoblotting and immunofluorescence analysis, we used mouse monoclonal antibodies against α-tubulin (DM1A; Sigma), FLAG-tag (M2; Sigma), Strep-tag II (4F1; MBL), GFP (JL-8; TaKaRa), and ALIX (sc-53540; Santa Cruz Biotechnology); rabbit polyclonal antibody against FLAG-tag (F7425; Sigma), Lamin B1 (ab16048, Abcam), and CHMP4B (ab105767; Abcam). Monoclonal antibodies to gQ1 (AgQ1-119) and rabbit polyclonal antibody against HHV-6B U14 were used as previously described (61, 62).

### Immunoblotting and immunofluorescence

Immunoblotting and immunofluorescence were performed as described previously (63, 64). For immunofluorescence, the cells were fixed with 4% paraformaldehyde and stained with the indicated antibodies. Nuclear DNA was stained with Hoechst 33342, and specific signals were detected using a confocal laser-scanning microscope (LSM800 microscope; Zeiss).

### Affinity precipitation

HEK293T cells were transfected with a plasmid expressing HHV-6A U34-Strep, Strep-HHV-6A U37, or an empty plasmid coupled with AcGFP-ALIX-NTD. After 48 hours, the cells were collected and lysed with 0.1% NP-40 buffer (50 mM Tris-HCl [pH 8.0], 150 mM NaCl, 0.5% NP-40) containing a protease inhibitor cocktail (Nacalai Tesque). After centrifugation, the supernatants were treated with MagStrep “type3” Strep-Tactin beads (IBA) with rotation for 2 hours at 4°C. The precipitates were collected by brief centrifugation, washed extensively with 0.1% NP-40 buffer, and analyzed by immunoblot.

### Calculation of virus genome copy numbers

A total of 2 × 10^5^ cells (JJhan) were infected with U1102 (1 × 10^7^ genome copies). After 72 hours, viral DNA was extracted from the supernatant of the cells using the DNeasy Blood & Tissue Kit (QIAGEN). The genome copy number per milliliter of infected cells was quantified by qPCR using SYBR Select master mix (Thermo Fisher Scientific). The sequences of primers used for this purpose were 5ʹ-CGCTAGGTTGAGAATGATCGA-3ʹ (forward) and 5ʹ-CAAAGCCAAATTATCCAGAGCG-3ʹ (reverse) as described previously (56). For membrane fractionation assays, the cells were lysed and fractionated using the LysoPure Nuclear and Cytoplasmic Extractor Kit (Fuji Wako) according to the manufacturer’s protocol.

### Stapled peptide

To impair ALIX-CHMP4 interactions, stapled peptide that mimics the ALIX binding helix in CHMP4A residues 205-222 (PKVDEDEEALKQLAEWVS), conjugated with octa-arginine at its C-terminus (46) was purchased from Peptide Institute Inc. Briefly, E212 and E219 in CHMP4A_205-222_ were replaced with (R)-4-pentenylAla and (S)-7-octenylAla, respectively, conjugated to form stapled peptide and dissolved in DMSO (Fig. 9A). To analyze the effect of this peptide on ALIX and CHMP4B interactions, HEK293T cells were transfected with AcGFP-ALIX-NTD and TagRFP-CHMP4B and incubated with 20 µM of CHMP4A_205-222_ for 3 days. These cells were analyzed by confocal microscopy. To analyze the effect of CHMP4A_205-222_ on viral infection, JJhan cells were infected with HHV-6A U1102 as described above, followed by incubation with DMSO or 20 µM of CHMP4A_205-222_ or mock-treated for 72 hours.

### Cell viability determination

A total of 1 × 10^4^ cells were cultured in 96-well plates in the presence or absence of 1 µg/mL Dox or 20 µM CHMP4A_205-222_ for 72 hours. Three hours prior to determining cell viability, 10 µL of Cell Counting Kit-8 solution (Dojindo) was added. The amount of formazan dye generated by the dehydrogenase in living cells was determined by measuring the absorbance at 450 nm using a microplate reader, following the manufacturer’s instructions.

### Ratio of multinucleated or fragmented nuclei

A total of 1 × 10^5^ cells were cultured in the presence of 1 µg/mL Dox for 72 hours. Nuclear DNA was stained with Hoechst 33342, and specific signals were detected using a confocal laser-scanning microscope. The ratio was calculated as the number of cells exhibiting multinucleated or fragmented nuclei among EGFP-positive cells. At least 44 cells were counted per sample.

### Statistical analysis

For comparisons of two groups, statistical analysis was performed using the unpaired Student’s *t*-test. Tukey’s test was used for multiple comparisons. A *P*-value >0.05 was considered not significant (n.s.).

## Data Availability

No data sets or code were generated or analyzed during the current study.

## AFTER EPUB

*[This article was published on 19 August 2025 with an error in Fig. 12. The figure was updated in the current version, posted on 20 August 2025.]*
